# A Facile Method to Probe the Vascular Permeability of Nanoparticles in Nanomedicine Applications

**DOI:** 10.1038/s41598-017-00750-3

**Published:** 2017-03-31

**Authors:** Yan Teck Ho, Giulia Adriani, Sebastian Beyer, Phan-Thien Nhan, Roger D. Kamm, James Chen Yong Kah

**Affiliations:** 1 NUS Graduate School for Integrative Sciences and Engineering, National University of Singapore, Singapore, Singapore; 2 BioSyM Interdisciplinary Research Group, Singapore-MIT Alliance for Research and Technology, Singapore, Singapore; 3 Federal Institute for Materials Research and Testing, Germany, Germany; 4 Department of Mechanical Engineering, National University of Singapore, Singapore, Singapore; 5 Department of Biological Engineering and Department of Mechanical Engineering, Massachusetts Institute of Technology, Massachusetts, USA; 6 Department of Biomedical Engineering, National University of Singapore, Singapore, Singapore

## Abstract

The effectiveness of nanoparticles (NP) in nanomedicine depends on their ability to extravasate from vasculature towards the target tissue. This is determined by their permeability across the endothelial barrier. Unfortunately, a quantitative study of the diffusion permeability coefficients (P_d_) of NPs is difficult with *in vivo* models. Here, we utilize a relevant model of vascular-tissue interface with tunable endothelial permeability *in vitro* based on microfluidics. Human umbilical vein endothelial cells (HUVECs) grown in microfluidic devices were treated with Angiopoietin 1 and cyclic adenosine monophosphate (cAMP) to vary the P_d_ of the HUVECs monolayer towards fluorescent polystyrene NPs (pNPs) of different sizes, which was determined from image analysis of their fluorescence intensity when diffusing across the monolayer. Using 70 kDa dextran as a probe, untreated HUVECs yielded a P_d_ that approximated tumor vasculature while HUVECs treated with 25 μg/mL cAMP had P_d_ that approximated healthy vasculature *in vivo*. As the size of pNPs increased, its P_d_ decreased in tumor vasculature, but remained largely unchanged in healthy vasculature, demonstrating a trend similar to tumor selectivity for smaller NPs. This microfluidic model of vascular-tissue interface can be used in any laboratory to perform quantitative assessment of the tumor selectivity of nanomedicine-based systems.

## Introduction

Nanoparticles (NPs) are widely studied as drug delivery vehicles to maximize drug efficacy through effective targeting of diseased tissue^1–4^. The most common delivery route of NPs is through the blood stream. Before NPs can reach the target tissue, they need to escape vascular flow through the fluid dynamic process of margination towards the vascular walls, adhere to the vascular endothelium, and extravasate across the endothelial cell (EC) barrier into the target tissue. This is true for both active^5, 6^ and passive targeting of NPs^7^.

Therefore, the effectiveness of NP-based drug delivery systems depends on their ability to extravasate into the target tissue. This is determined by the permeability of the endothelial barrier to the NPs which is in turn dependent on different physical attributes of the NPs. Unlike the abundance of studies that characterize cell adhesion and uptake of NPs with different shape, size and surface functionalities^8–11^, much less is known about how these attributes affect NPs extravasation. A systematic quantitative study of the permeability coefficients of the endothelium to NPs exiting the vasculature would enable a better understanding of how different physical characteristics affect their extravasation, and eventually guide their rational design towards an effective payload delivery.

Although permeability assays utilizing animal models are the most physiologically relevant, permeability measurements with *in vivo* or *ex vivo* systems often involve tedious and delicate creation of an experimental viewing window of the vasculature within the animal model^12, 13^. This is before intravenous introduction of a fluorescent tracer into the animal through a cannulated vein for probing permeability from a chosen blood vessel, which may not end up being suitable for observation^13^. A systematic quantitative study is therefore difficult to implement *in vivo* due to its complexity, high cost, heterogeneity, and consequently large variability in results; thus the limited studies on NPs extravasation in animal models.

Conventional *in vitro* assays have also been used to characterize permeability, most notably the use of multi-well plates fitted with additional membrane inserts such as the Boyden chamber or transwell membrane^14^. Whilst tunable, this technique provides only relative permeabilities against a reference instead of an absolute quantitative diffusional permeability coefficient^15–19^. Furthermore, such a technique does not allow for permeability measurements under dynamic flow conditions similar to that found in endogenous vasculature.

Computational models have also been developed for predicting the extravasation, accumulation, and delivery of NPs in tumors^20–22^. While these models are useful for predicting NP drug delivery behavior, efficacy, and efficiency upon administration, they do not fully account for the intricacies of trans-endothelial transport^23, 24^ that is highly dependent not just on the cell type, but also NP parameters such as shape and surface chemistry^25–27^. *In vitro* or *in vivo* permeability models would still be required to correlate computational data.

More recent attempts to study permeability using microfluidic channels formed in gels from extracellular matrix (ECM) proteins and collagen containing endothelial cells^28^ allowed for quantitative permeability measurements under flow conditions *in vitro* ^29–31^. However, these gel platforms are susceptible to deformation and collapse^28^. They are also relatively complex to fabricate compared to microfluidic devices made from polydimethylsiloxane (PDMS) via replica molding.

More recently, Kim *et al*. developed a two layered microfluidic device comprised of a microporous polyester membrane sandwiched between two independent microfluidic channels^32^. The system was similar to conventional transwell membrane assays where a fluorescent material can be added to the upper chamber of the device and allowed to diffuse across the permeable membrane into the lower chamber^32^. Kim *et al*. demonstrated tunable endothelial permeability with tumor necrosis factor alpha (TNF-α) treatment to probe NP translocation across the polyester membrane^32, 33^. The permeability of the membrane was then determined by measuring the fluorescence intensity in the bottom chamber^32, 34^ relative to a control in the top chamber after a fixed time interval. This relative intensity then provided a dimensionless quantitative measure of permeability.

Here, we describe a facile method to probe the vascular permeability of NPs by utilizing a relevant model of the vascular-tissue interface with tunable endothelial permeability *in vitro* based on PDMS microfluidics, and using a different approach based on an image analysis technique to extract the diffusional permeability coefficient (P_d_) of the endothelial barrier towards an analyte. ECs were cultured in a monolayer that mimicked the vasculature to allow for quantitative assessment of permeability measurements in units of cm/s determined by continuous imaging with standard fluorescence microscopy. We demonstrated tunability of the vascular permeability through treatment of ECs with angiopoietin 1 (Ang-1) and a cell membrane permeable form of cyclic adenosine monophosphate (pCPT-cAMP) to approximate both healthy and tumor vascular permeability. By applying image analysis on their fluorescent intensity, we were able to determine the P_d_ of fluorescent polystyrene NPs (pNPs) of different sizes when diffusing across the EC monolayer, which have not been demonstrated in other microfluidic devices to date.

## Materials and Methods

### Fabrication of PDMS microfluidic devices

**Figure 1:**
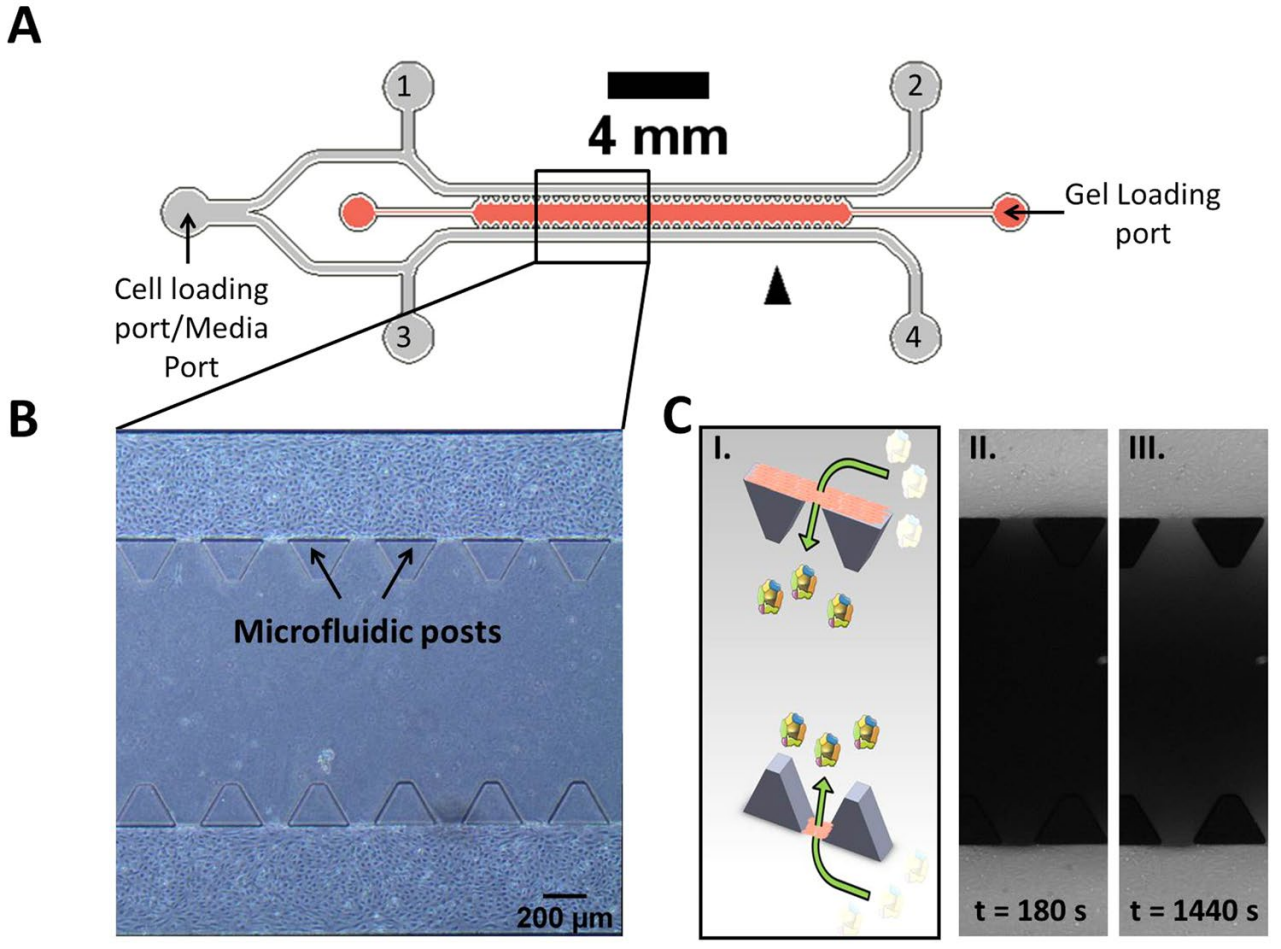
(**A**) Schematic of the microfluidic device used for *in vitro* probing of barrier permeability to NPs. (**B**) Depiction of untreated cells after 4 days in culture within the microfluidic device. The endothelial barrier through which the permeability is measured is located in between the microfluidic posts but is difficult to identify since it is perpendicular to the optical plane. (**C**) (I) Paned view, cross sectional depiction of the interface through which the diffusion of nanoparticles occurs. Nanoparticles flow via convection through the media channels before diffusing across a confluent, seeded endothelial monolayer in between two microfluidic posts into the central gel region. NPs in white indicate those that have yet to move across the endothelial barrier into the gel region of the microfluidic device. (II) Experimental illustration of the diffusion of fluorescent material into the central gel region 180 s into the experiment. (III) Experimental illustration of the diffusion of fluorescent material into the central gel region 1440 s into the experiment.

PDMS microfluidic devices consisting of a single gel channel flanked by two cell channels as were fabricated as shown in Fig. 1. The design of this microfluidic device was previously described in another study by the co-authors^35^. Instead of a one-channel device, having two opposite cell channels sandwiching a middle gel region in a 2-channel device would allow access to more data points for the same field of view captured by the fluorescence camera, thus increasing the throughput of the device without complicating the device fabrication process.

The devices were fabricated using standard soft lithography techniques^36, 37^; 10 units of silicone elastomer base were mixed with 1 unit of curing agent (Sylgard 184 silicone elastomer kit, Dow Corning, Midlands, USA), degassed, and allowed to polymerize on a silicon mold at 80 °C for 3 h. Cured PDMS replicas were cut into single devices, sonicated in 70% ethanol for 30 min and allowed to dry at 80 °C overnight. The devices were then plasma bonded onto glass cover slips (VWR international, Pennsylvania, USA), and incubated with 1 mg/mL Poly-D-lysine (PDL) (Sigma Aldrich, Missouri, USA) into the channels overnight at 4 °C. PDL coated devices were then washed with Milli-Q water before being dried at 70 °C for 24 h to restore hydrophobicity and stored at 4 °C until cell seeding.

### Preparation of Dextran solutions

Oregon Green 488 tagged 10 kDa (wavelength_excitation_/wavelength_emission_, λ_ex_/λ_em_ = 501 /526 nm) and Texas Red tagged 70 kDa Dextran (λ_ex_/λ_em_ = 561 nm/ 594 nm) (Life Technologies, Massachusetts, USA) were reconstituted in endothelial growth medium (EGM-2, Lonza, Basel, Switzerland) at a concentration of 25 µg/mL before each experiment.

### Preparation and characterization of polystyrene nanoparticles

Fluorescent carboxylate pNPs sized 20, 40, 100, 200 nm (F-8786, F-8793, F-8801, F-8763, FluoSpheres, Life Technologies, USA) (λ_ex_/λ_em_ = 580/605 nm) were reconstituted in 100% Fetal Bovine Serum (FBS) (16000–044, Gibco, USA) overnight at 4 °C at a concentration of 5 × 10^12^ beads/mL to form a protein corona and maintain their colloidal stability. All corona coated pNPs were freshly prepared before each experiment and diluted 10 times in EGM-2 to a final concentration of 5 × 10^11^ beads/mL prior to permeability experiments. The hydrodynamic diameter, *D*_*H*_ of different pNPs was measured using dynamic light scattering (DLS) (Nano ZS, Malvern, UK).

### Human umbilical vein endothelial cell culture

Human umbilical vein endothelial cells (HUVECs) (Lonza, Basel, Switzerland) were chosen as the model endothelial barrier in this study. The HUVECs were expanded and passaged 5 times (P + 5) before being introduced into the devices. All HUVECs were cultured in EGM-2 (Lonza, Basel, Switzerland) and trypsinized (Lonza, Basel, Switzerland) when ~70–80% confluent before being seeded into the microfluidic devices. Cells were cultured in incubators at 37 °C and 5% CO_2_. All HUVECs were purchased commercially and all methods and experimental protocols involving HUVECs in this work were conducted with protocols in accordance with relevant guidelines and regulations stipulated and approved by the Singapore MIT alliance for Research and Technology’s review board.

### Gel filling and seeding of HUVECs into the microfluidic devices

PDMS microfluidic devices were sterilized with UV irradiation for 25 min prior to cell seeding. Fibrin solution was then prepared by mixing 5 mg/mL fibrinogen from bovine plasma (Sigma Aldrich, Missouri, USA) in 1x phosphate buffer saline (PBS) with 1.24 units/mL of thrombin in 1x PBS (Sigma Aldrich, Missouri, USA) in a 1:1 ratio. The middle gel region of the microfluidic devices was filled with 7 µL of the fibrin solution via the gel loading port (Fig. 1A) and allowed to polymerize at 37 °C for 30 min to form a gel. Based on images of fluorescently tagged fibrin gel, we obtained an approximate pore sizes ranging from ≈6 to 9 µm which were much larger than the largest 200 nm pNPs used in this study (data not shown), thus suggesting that the pore size of the fibrin gel would not limit the diffusion of pNPs in the central gel region and thereby affecting the calculated P_d_.

After gelation, the side media channels were incubated with 50 µg/mL fibronectin from human plasma (Sigma Aldrich, Missouri, USA) dissolved in EGM-2 for 1 h at 37 °C and 5% CO_2_ to provide a conducive surface for HUVEC attachment. This is followed by the addition of 30 µL of cell suspension to the cell loading port (Fig. 1A). The seeding concentration varied with different treatments. Untreated and Ang-1 treated devices were seeded with 10 × 10^6^ cells/mL whereas pCPT-cAMP treated devices were seeded at a lower concentration of 5 × 10^6^ cells/mL because they proliferated much faster than in the untreated case. We also observed that with these cells seeding concentrations, a confluent monolayer would form within the microfluidic device four days after seeding.

After cell seeding, the devices were incubated at 37 °C, 5% CO_2_ for 4 h to allow for cell attachment before replacing the culture medium within the cell-seeded channel with 120 µL of fresh medium. Cell culture medium was subsequently changed every 24 h. The cells were cultured to confluence in order to form a complete monolayer at the gel interface by day 4. The devices were then kept for an additional day to allow them to stabilize before experiments were conducted on days 5 and 6. All devices were checked for a complete HUVEC monolayer under phase contrast microscopy before the permeability measurements (Fig. 1B).

### Ang-1 and cAMP treatment to tune vascular permeability

For Ang-1 (R&D Systems, Minnesota, USA) treatment, seeded HUVECs were cultured in the device with EGM-2 until confluence and treated for 24 h with Ang-1 in EGM-2 at four concentrations: 100, 300, 500 and 5000 ng/mL before the permeability experiments. For pCPT-cAMP (Sigma-Aldrich, Missouri, USA) treatment, HUVECs were treated starting 4 h after the cell seeding at five concentrations: 0.5, 25, 50, 200, and 250 μg/mL, and then cultured with EGM-2 containing pCPT-cAMP until confluence.

### Permeability assay

The method for quantifying diffusional permeability in our PDMS microfluidic devices was modified based on first principles from a previously established protocol taking into account the differences in channel geometry between the rectangular lumen of the microfluidic device used in this study and circular lumen used in the previous study^31^. Prior to the experiments, the cell culture medium was aspirated from all media ports within the device. The device was then placed on the stage of an Olympus IX81 inverted microscope (Olympus, Tokyo, Japan). Fluorescence images were acquired to determine the background intensity. 15 μL of fluorescent dextran (both 10 kDa and 70 kDa as described earlier) or pNPs was then added through the media port and allowed to completely fill the HUVEC channels before starting the time lapse image acquisition at intervals of 30 s to observe the diffusion of fluorescent material across the endothelial barrier into the gel region (Fig. 1C). The total acquisition time was 30 min for dextran and 45 min for pNPs due to their slower diffusion.

We note that the presence of noise in the image acquisition and analysis process may introduce inaccuracies to the P_d_ obtained. Price and Tien *et al*.^31^ suggest that at low permeability, the change in fluorescence intensity with time in the middle gel region is low, and therefore susceptible to artefacts arising from low signal-to-noise ratio, leading to potentially inaccurate P_d_ values. Here, we maximize the signal-to-noise ratio by using an electron multiplying CCD camera (Andor iXon^+^ EMCCD camera, Andor, Belfast, Northern Ireland) with a sensitivity capable of measuring single photons to reduce the possibility of artefacts for low permeability barriers.

### Image analysis to determine diffusional permeability

**Figure 2:**
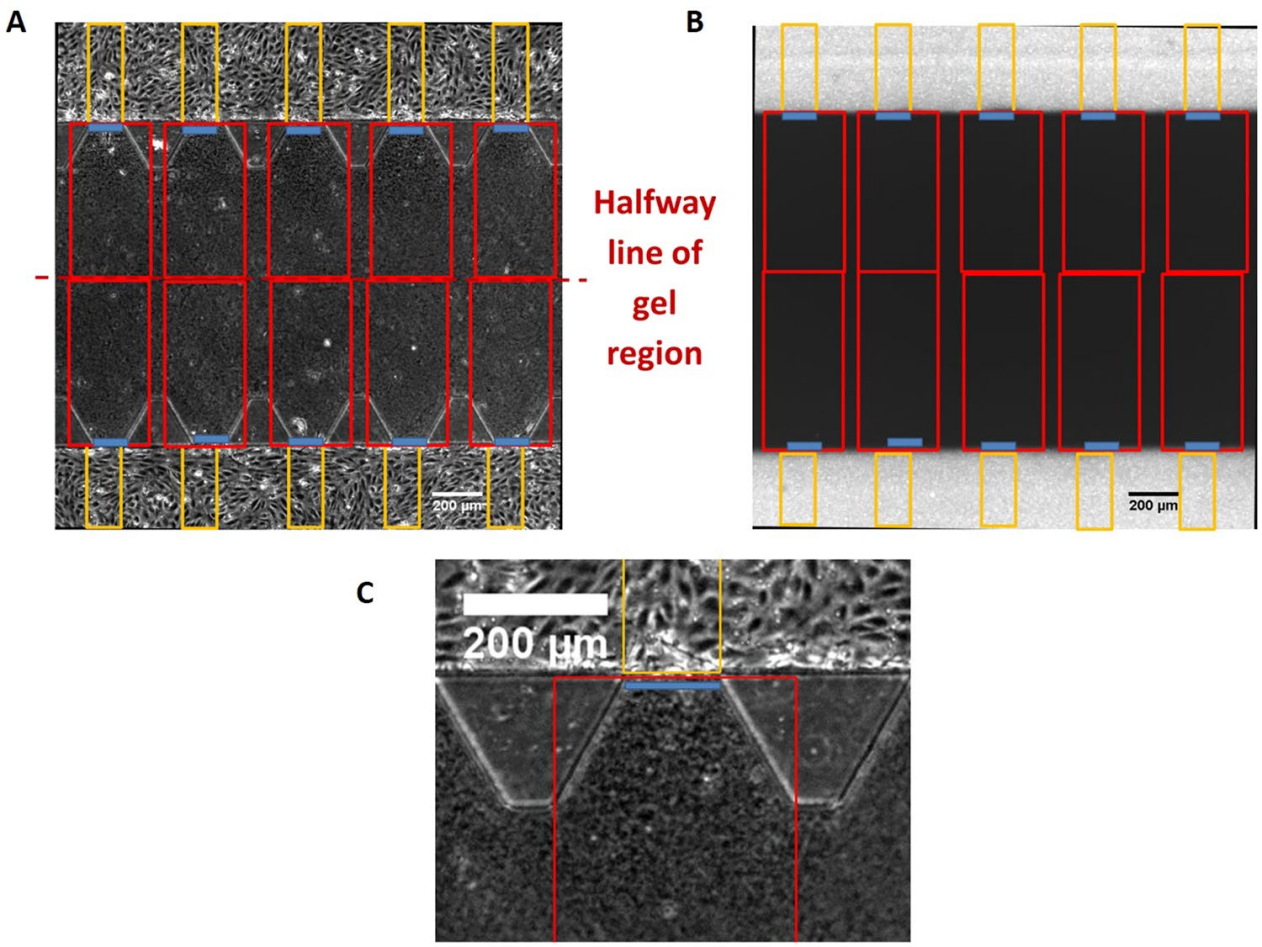
Representative images illustrating the different regions of interest (ROIs) used in the data analysis to calculate the permeability. (**A**) *ROIs* demarcated by MATLAB illustrated on a phase-contrast image. A single set of yellow, blue, and red ROIs yields a single data point. (**B**) *ROIs* on the same field of view of a representative fluorescent image from the flow experiments at time t = 0, which is denoted as the time in which the fluorescent material completely fills the lumen of the HUVEC channels before diffusing into the gel region. (**C**) A magnified view of the *ROI* in the green oval to show the boundaries of the different regions from which intensity values are extracted. The boundary of the red box lies completely within the gel region with the blue line flush to the endothelial monolayer whilst being also within the red box. The yellow box delineates the region within the luminal space of the endothelial monolayer.

The time lapse images were analyzed by dividing the field of view into different regions of interest (ROI) (as delineated by different colors in Fig. 2A and B, with the green oval delineating a single data point from a single device). At time t = 0 s, the dextran or pNPs-containing solution filled the lumen of the HUVEC side channels before diffusing into the middle gel region (Fig. 2B). The average intensity values were extracted from each ROI using MATLAB and then used to calculate the P_d_ as described below. Data points arising from obvious focal leaks within the HUVEC monolayer were omitted. Focal leaks were defined based on the method previously described^31^. Background fluorescent intensity was subtracted from all fluorescent intensity values used in the calculation.

In calculating the diffusional permeability, we assumed negligible convective contribution to transport since care was taken to ensure that no pressure difference existed between the two fluid-filled channels. The transport of solute across the endothelial monolayer was therefore attributed solely to static diffusion. The P_d_, when the lumen was completely filled with solute, was then defined as^31, 33^:

1 $${P}_{d}=\frac{{J}_{s}}{{A}_{monolayer}\cdot {\rm{\Delta }}C}$$

Here J_s_ refers to the diffusion flux of solute across the endothelial monolayer; A_monolayer_, the surface area of the monolayer where the diffusion occurs (delineated blue in Fig. 2C); and ΔC, the concentration difference of the diffusing solute across the endothelial monolayer. From mass conservation, J_s_ can be defined as the rate of change in number of diffusing NPs, N, within the gel region (red box in Fig. 2B and C):

2 $${J}_{s}=\frac{dN}{d{t}_{gel}}=\frac{d}{dt}{\int }_{gel}CdV$$

where C denotes the local solute concentration in number of NPs per unit volume, and dV, an infinitesimal volume within the gel region. Considering C being directly proportional to its fluorescence intensity, I, and substituting Equation (2) into (1):

3 $${P}_{d}=\frac{\frac{d}{dt}{\int }_{gel}IdV}{{A}_{monolayer}\cdot \le {\rm{\Delta }}I}\,$$

Given ${I}_{ave,gel}=\frac{1}{{V}_{gel}}{\int }_{gel}IdV$, Equation (3) becomes:

4 $${P}_{d}=\frac{\frac{d}{dt}({I}_{ave,gel}\cdot {V}_{gel})}{{A}_{monolayer}\cdot {({I}_{lumen}-{I}_{y=0})}_{t=0}}=\frac{{A}_{gel}\cdot (\frac{d{I}_{ave,gel}}{dt})}{{w}_{monolayer}\cdot {({I}_{lumen}-{I}_{y=0})}_{t=0}}$$

where A_gel_ refers to the surface area of the red ROI within the gel; w_monolayer_ is the width of the HUVEC monolayer across which diffusion occurs ${(\frac{d{I}_{ave,gel}}{dt})}_{t=0}$; is the rate of change of average fluorescence intensity within the red ROI in Fig. 2B at time t=0; I_lumen_ is the average intensity of fluorescence in the HUVEC channel (demarcated as yellow ROIs in Fig. 2B); and I_y=0_ is the average local intensity one pixel wide within the gel adjacent to the HUVEC monolayer (indicated by the blue ROI in Fig. 2C), both at time t = 0.

Since larger nanoparticles or diffusing materials would experience slower diffusion within the gel region, it is imperative that the control volume within the gel region (demarcated by the red ROI within the gel in Fig. 2) includes the interface between the HUVEC monolayer and the gel (blue ROI in Fig. 2), so that any changes in fluorescence intensity due to the slow diffusion and accumulation of larger materials within the gel region would still be accounted for in quantifying its P_d_.

### Immunostaining and confocal imaging

HUVECs were fixed with 4% paraformaldehyde (PFA) (Sigma Aldrich, USA), permeabilized with 0.1% Triton X (Sigma Aldrich, Missouri, USA) and blocked with 3% bovine serum albumin (BSA) (Sigma Aldrich, Missouri, USA) reconstituted in 1x PBS for 3 h at room temperature. The microfluidic devices were then incubated with either rabbit anti-VE-Cadherin (Enzo life sciences, New York, USA) or mouse anti-ZO-1 (Life Technologies, Massachusetts, USA) primary antibodies overnight at 4 °C, followed by Alexa Fluor 488 chicken anti-rabbit (Life Technologies, Massachusetts, USA) or goat anti-mouse (Life Technologies, Massachusetts, USA) secondary antibodies for VE-Cadherin and ZO-1 staining, respectively. Hoescht (Life Technologies, Massachusetts, USA) and Rhodamine Phalloidin (Life Technologies, Massachusetts, USA) were used to stain the nuclei and F-actin cytoskeleton of HUVECs, respectively. The microfluidic devices were then imaged under an Olympus IX83 confocal microscope (Olympus, Tokyo, Japan) for a cross sectional z-stack of the HUVEC channel.

## Results and Discussion

### Diffusional permeability coefficients of dextran across HUVEC monolayers

**Figure 3:**
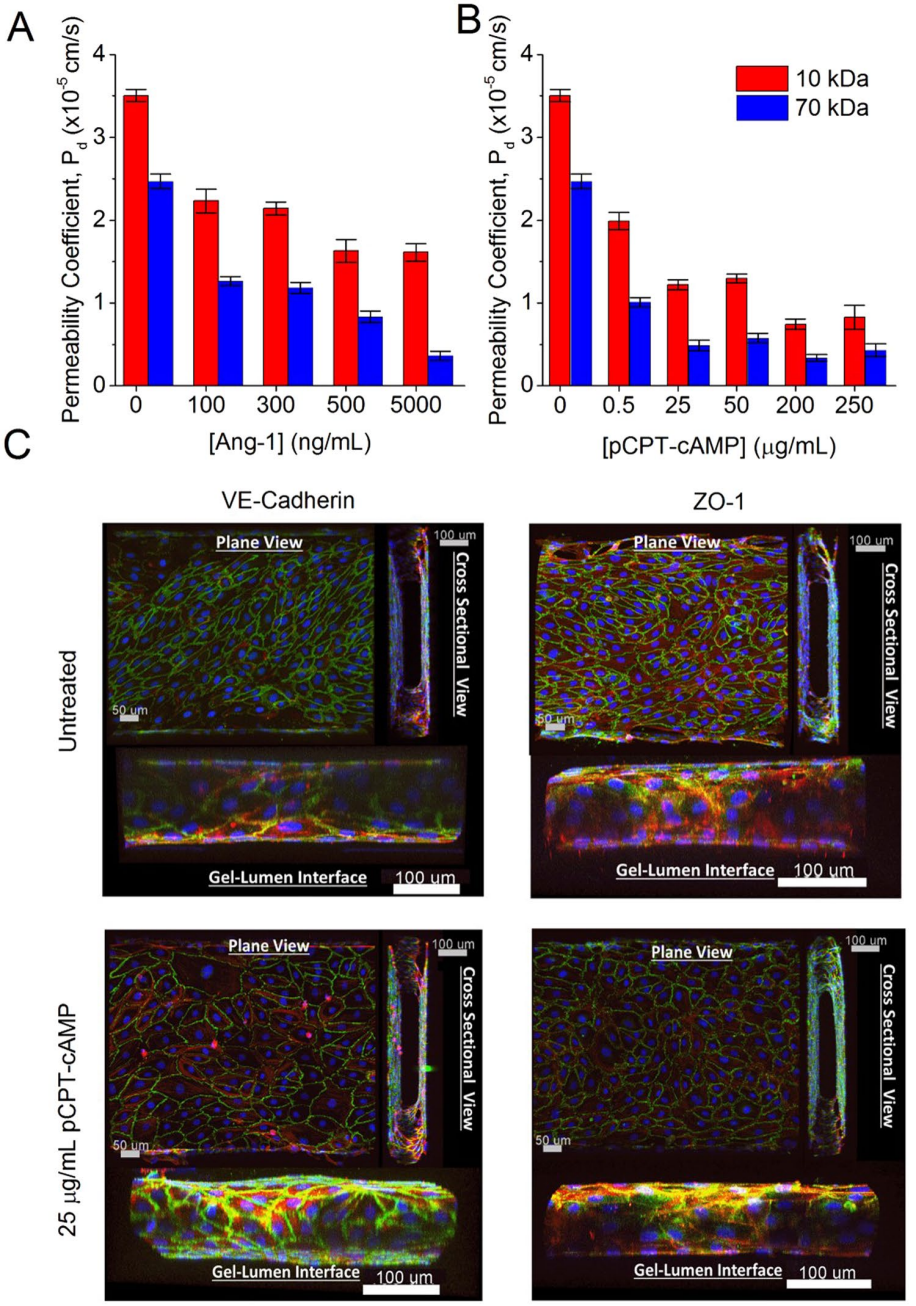
Titration experiments with HUVECs treated with different concentrations of Ang-1 and pCPT-cAMP. (**A**) Graph showing calculated permeability coefficients for 10 kDa and 70 kDa dextrans with Ang-1 treatment. Data show mean values and SEM. There is no statistical significant difference in P_d_ values for 10 kDa between 100 and 300 ng/mL Ang-1 treated HUVECs, and 500 and 5000 ng/mL Ang-1 treated HUVECs. In the case of 70 kDa dextran, all results are statistically significant (Student’s t-test *p < 0.05) except between 100 and 300 ng/mL Ang-1 treated HUVECs. (**B**) Graph showing calculated permeability coefficients for 10 kDa and 70 kDa dextrans with pCPT-cAMP treatment. Data show mean values and SEM. In the case of 10 kDa dextran, P_d_ values are statistically different (Student’s t-test *p < 0.05) except between 25 and 50 µg/mL pCPT-cAMP treated HUVECs, and 200 and 250 µg/mL pCPT-cAMP treated HUVECs. In the case for 70 kDa dextran, all P_d_ values recorded are statistically different except between 25, 50, 200, and 250 µg/mL pCPT-cAMP treated HUVECs (Student’s t-test. *p < 0.05). (**C**) Representative confocal images of HUVECs stained for adherens junction protein VE-Cadherin and tight junction protein ZO-1 for 25 µg/mL pCPT-cAMP treated and untreated cells. Green (Alexa Fluor 488) stains for the adherens and tight junction proteins VE-Cadherin and ZO-1; red (Rhodamine Phalloidin) stains for actin; and in blue (Hoescht) are the cell nuclei.

Untreated HUVEC monolayers were first characterized for their diffusional permeability coefficients, P_d_ to both 10 kDa and 70 kDa dextrans. The measurements showed size-selectivity as observed in vascular networks *in vivo* ^38–40^, with a mean P_d_ of the smaller-sized molecules (10 kDa dextran, P_d_ = 3.50 × 10^−5^ cm/s) being significantly higher (p ≤ 0.0001) than the mean P_d_ of the larger-sized molecules (70 kDa dextran, P_d_ = 2.47 × 10^−5^ cm/s) (Fig. 3A, with no Ang-1 or pCPT-cAMP). These values of P_d_ obtained with 10 and 70 kDa dextran were comparable to the values reported with 40 kDa dextran on untreated HUVEC monolayers in conventional transwell assays^41^. These observations suggest that the HUVEC monolayers form slightly tighter para-cellular junctions in our microfluidic devices compared to conventional two component transwell permeability assays, thus resulting in the slightly lower P_d_ values even for smaller sized 10 kDa dextran.

While these P_d_ values obtained *in vitro* agree with those previously reported in other microfluidic *in vitro* systems^42, 43^, they were significantly higher than the P_d_ values in healthy vasculature *in vivo*. In the microvessels of mammalian skeletal muscles, 10 kDa and 70 kDa dextran were reported to have a P_d_ in the range of ~10^−6^ cm/s and 10^−7^ cm/s, respectively^38, 44^. The thickness of the HUVEC monolayer could influence the P_d_ values measured. Physiologically, endothelial cells vary in thickness depending on the vessel type and location, with cell thickness ranging from ≈0.1 µm for capillaries and veins, to 1 µm for aortas^45^. Based on the thickness of the fluorescent cross section of the monolayer in our confocal images (Fig. 3C), we determined our HUVEC cell thickness to be ≈2 µm, comparable to values expected of human endothelial cells.

However, the human vasculature consists of multiple supporting stromal cells such as fibroblasts, vascular smooth muscle cells and pericytes working in concert with the parenchymal epithelial cells to regulate vascular permeability. The significantly lower permeability *in vivo* could be attributed to tighter cell-cell junctions and/or the presence of these multiple supporting cells that were not present in the *in vitro* assays, which consisted of a mono-culture of ECs. These various supporting cells work in concert with ECs to maintain and regulate vascular integrity and permeability through a variety of mechanisms^46–48^.

In contrast, the P_d_ values of dextran we obtained are closer to tumor vascular permeability, which is known to be leakier compared to healthy vessels^49, 50^. Previous studies showed that the P_d_ of 10 kDa and 70 kDa dextran across murine tumor vasculature ranged between 1 and 3 × 10^−6^ cm/s, with 10 kDa expectedly having a higher P_d_ than 70 kDa in these tumor models^13, 42^.

Here, we tuned the vascular diffusional permeability coefficient to attain values closer to physiologically healthy levels by treating the ECs in the microfluidic device with both Ang-1 and pCPT-cAMP. This allows us to maintain an easy-to-handle system and to obtain relevant physiological response from the *in vitro* model despite phenotypical differences. Furthermore, the tuning of permeability by cytokines introduced an additional level of control of the system that allows systematic permeability studies over a range of vasculature permeabilities, lending versatility to the microfluidic model.

### Tuning vascular permeability with Ang-1

Treatment of confluent HUVECs with different concentrations of Ang-1 for 24 h led to a concentration dependent decrease in P_d_ when probed with both 10 kDa and 70 kDa dextran (Fig. 3A). For 10 kDa dextran, the P_d_ decreased 1.57 and 2.17-fold with 100 ng/mL and 5 µg/mL treatment of Ang-1 to 2.23 × 10^−5^ cm/s and 1.61 × 10^−5^ cm/s respectively. For 70 kDa dextran, the P_d_ decreased 1.94 and 6.86-fold to 1.27 × 10^−5^ cm/s and 3.60 × 10^−6^ cm/s with 100 ng/mL and 5 µg/mL of Ang-1, respectively.

Ang-1 is a cytokine known to reduce vascular permeability through several mechanisms^15–17, 51, 52^. First, Ang-1 binds antagonistically with Ang-2 to tyrosine kinase receptor (Tie-2) to promote vascular maturation and stabilization^53, 54^. It also modulates the increase in vascular permeability due to other growth factors such as VEGF, thrombin, histamine, and bradykinin^55^ through inhibiting the c-Src pathway that leads to the phosphorylation and eventual internalization of VE-Cadherin^17, 55^. Ang-1 also reduces vascular permeability by reducing the basal phosphorylation of PECAM-1^56^. In addition to maintaining the integrity of junctional proteins, Ang-1 signaling reduces intracellular Ca^2+^ concentration, which at high levels, is known to increase vascular permeability^57–59^.

In addition to the reduced permeability after Ang-1 treatment, an enhancement in size-selectivity was also observed, as the larger 70 kDa dextrans registered a significantly lower P_d_ value compared to their 10 kDa counterparts across all concentrations of Ang-1 (p < 0.05). The decrease in permeability with Ang-1 treatment appeared more pronounced for larger molecules, even though the P_d_ values of the endothelial monolayer for both sizes of dextran were still above physiological levels.

### Tuning vascular permeability with pCPT-cAMP

Similar to Ang-1, cAMP also reduces vascular paracellular permeability by promoting the expression of adherens and tight junction proteins such as ZO-1, VE-cadherin, claudin-5, and junction adhesion molecules (JAMs)^19, 39, 60–62^. Here, treatment with pCPT-cAMP resulted in a concentration dependent decrease in P_d_ across the ECs for both 10 kDa and 70 kDa dextran (Fig. 3B). While the P_d_ for 10 kDa dextran exhibited a 2.87-fold decrease to 1.22 × 10^−5^ cm/s with treatment of 25 µg/mL of pCPT-cAMP, the same treatment caused the P_d_ of 70 kDa dextran to decrease 5.05-fold to 4.88 × 10^−6^ cm/s.

As with Ang-1, we observed size selective permeability for all concentrations of pCPT-cAMP (70 kDa dextran having significantly lower P_d_ values compared to 10 kDa dextran, p < 0.05) and a more pronounced decrease in permeability for the larger molecular weight dextran with pCPT-cAMP treatment. Such size selectivity was in line with what is expected *in vivo* ^38^.

In addition to the differences in diffusion coefficients, the differences in P_d_ observed were also a result of different molecular weight dextrans experiencing different degrees of exclusion due to molecular size when passing through the endothelial barrier^12, 63^. Michel and Curry *et al*. observed that the ratio of a molecule’s P_d_ over its diffusivity, D i.e. P_d_/D followed a non-linear decline against the molecular radius^38, 64^. This suggests that the decrease in permeability may not be due solely to the decrease in diffusivity following the increase in the particle’s size, although the scatter in our data prevents us from drawing any definite conclusions on the role of exclusion. Therefore, additional effects such as increased viscous drag and an exclusion mechanism at the inter-endothelial junctions for larger sized particles moving across the endothelial barrier may also play a role in mediating the observed size-selective permeability^38^.

With increasing pCPT-cAMP concentrations that reduce paracellular permeability, the increase in viscous drag and exclusion experienced by larger sized 70 kDa dextran could, as a result, be more significant than the one for the smaller sized 10 kDa dextran. This may explain the more pronounced decrease in permeability for larger molecular weight dextran as pCPT-cAMP treatment concentrations increase.

Taken together these results show that untreated cells and 25 μg/mL pCPT-cAMP treated cells both resulted in P_d_ values an order higher than that reported *in vivo* for cancer and normal healthy vasculature, respectively. While this microfluidic model did not achieve the exact permeability values *in vivo*, it did provide approximate *in vivo* values that showed the right trend in permeability moving from a healthy to tumor vasculature. We can subsequently address the differences with an appropriate constant correction. Nonetheless, we would consider 25 µg/mL pCPT-cAMP treated devices and untreated devices to approximate normal and tumor vascular permeabilities respectively.

Confocal imaging of the endothelial monolayer in the microfluidic devices stained for adherens junction protein VE-cadherin and tight junction linker protein ZO-1 have demonstrated inter-endothelial adherens and gap junctions formation for both untreated and 25 μg/mL pCPT-cAMP-treated microfluidic devices (Fig. 3C). Interestingly, treatment of HUVECs with 25 μg/mL pCPT-cAMP resulted in a more localized expression of VE-cadherin compared to the untreated counterpart as indicated by the less “fuzzy”, “thinner” and more defined staining, even at the gel-lumen interface (Fig. 3C). Treatment by pCPT-cAMP also led to cells growing in a more ordered manner as compared to the untreated counterparts. These are characteristics of a decreased vascular permeability due to reduced angiogenic potential following treatment with pCPT-cAMP^65^. On the other hand, there was no observable difference in the ZO-1 staining between 25 μg/mL pCPT-cAMP treated and untreated cells.

### Characterization of polystyrene nanoparticles

**Figure 4:**
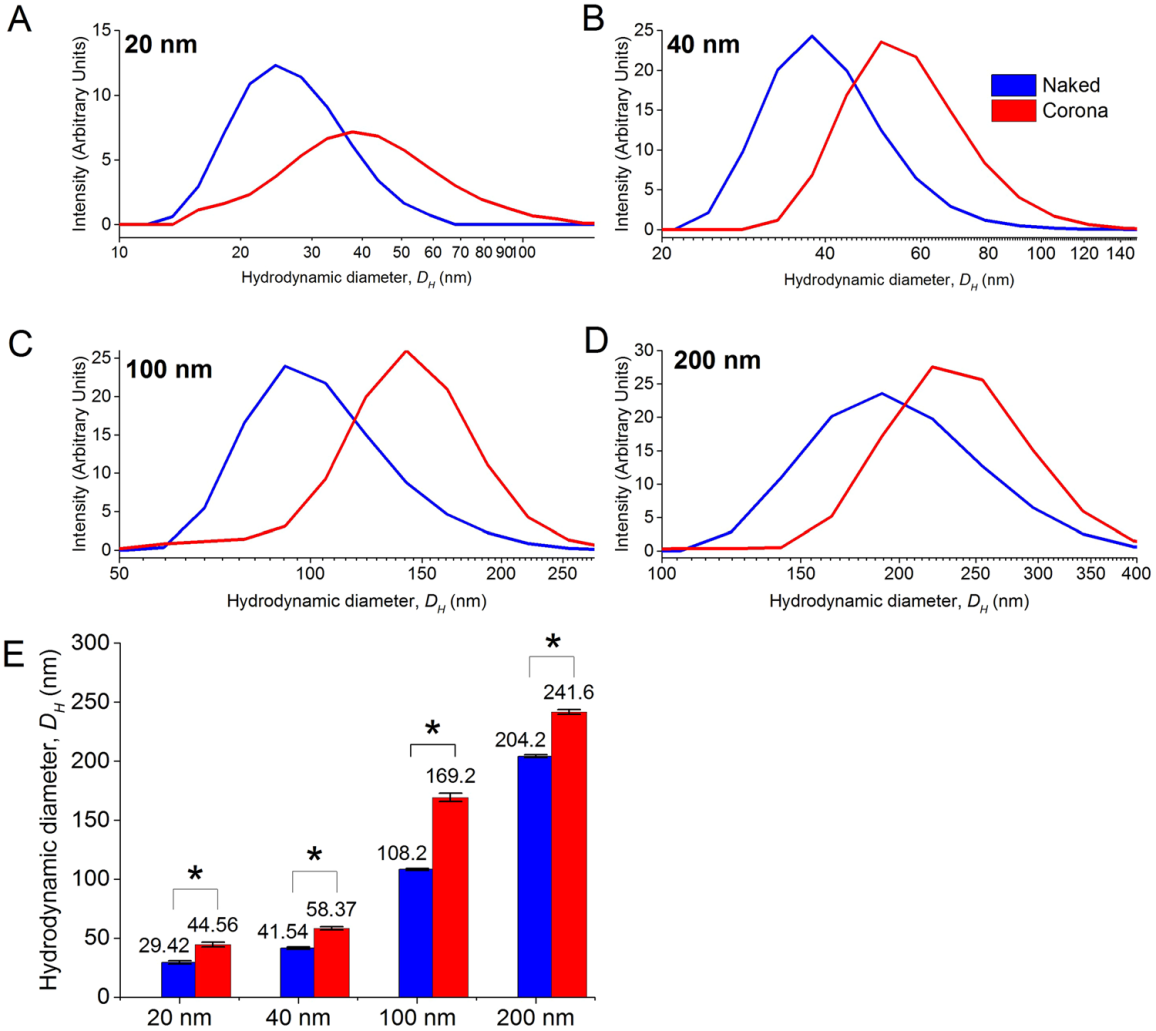
Characterization of size distribution of FluoSpheres carboxylate-modified polystyrene nanospheres (Life Technologies) with dynamic light scattering (DLS). (**A–D**) Size distribution plots for 20 nm, 40 nm, 100 nm, and 200 nm spheres with and without a corona, respectively. (**E**) Mean hydrodynamic diameter, *D*_*H*_ for 20 nm, 40 nm, 100 nm, and 200 nm spheres with (red) and without (blue) a protein corona formed from FBS. Student’s t-test. *p < 0.05.

The pNPs used in this study aggregated easily in cell culture medium. To maintain their colloidal stability, we reconstituted them in FBS to pre-form a protein corona coating around them. The average hydrodynamic diameter, *D*_*H*_ of all the pNPs increased by ~32 nm with the formation of a protein corona (Fig. 4). We observed a similar increase in the *D*_*H*_ previously upon formation of a protein corona^66, 67^. The formation of protein corona on all NPs in contact with biological media e.g. cell culture media and blood, and the consequent increase in *D*_*H*_ is inevitable. As such, we characterized the size change of the pNPs upon protein corona formation (Fig. 4) and reported subsequent P_d_ measurements of the pNPs according to their measured *D*_*H*_.

Since the increase in hydrodynamic diameter was consistent across all sizes of pNPs, this would not affect any size-dependent trend in their permeability caused by the increase in size due to the protein corona. Furthermore, while it is true that the protein corona may evolve with time due to protein exchanges between those on the pNP’s surface and the free proteins in the medium^68^, such an exchange is unlikely in our study as the pre-formed protein corona was formed from FBS following an overnight incubation before being flowed in EGM medium also containing FBS as the serum component so as to minimize this exchange. This ensured that the protein corona stayed relatively constant over the vascular flow.

### Permeability measurements for nanoparticles

**Figure 5:**
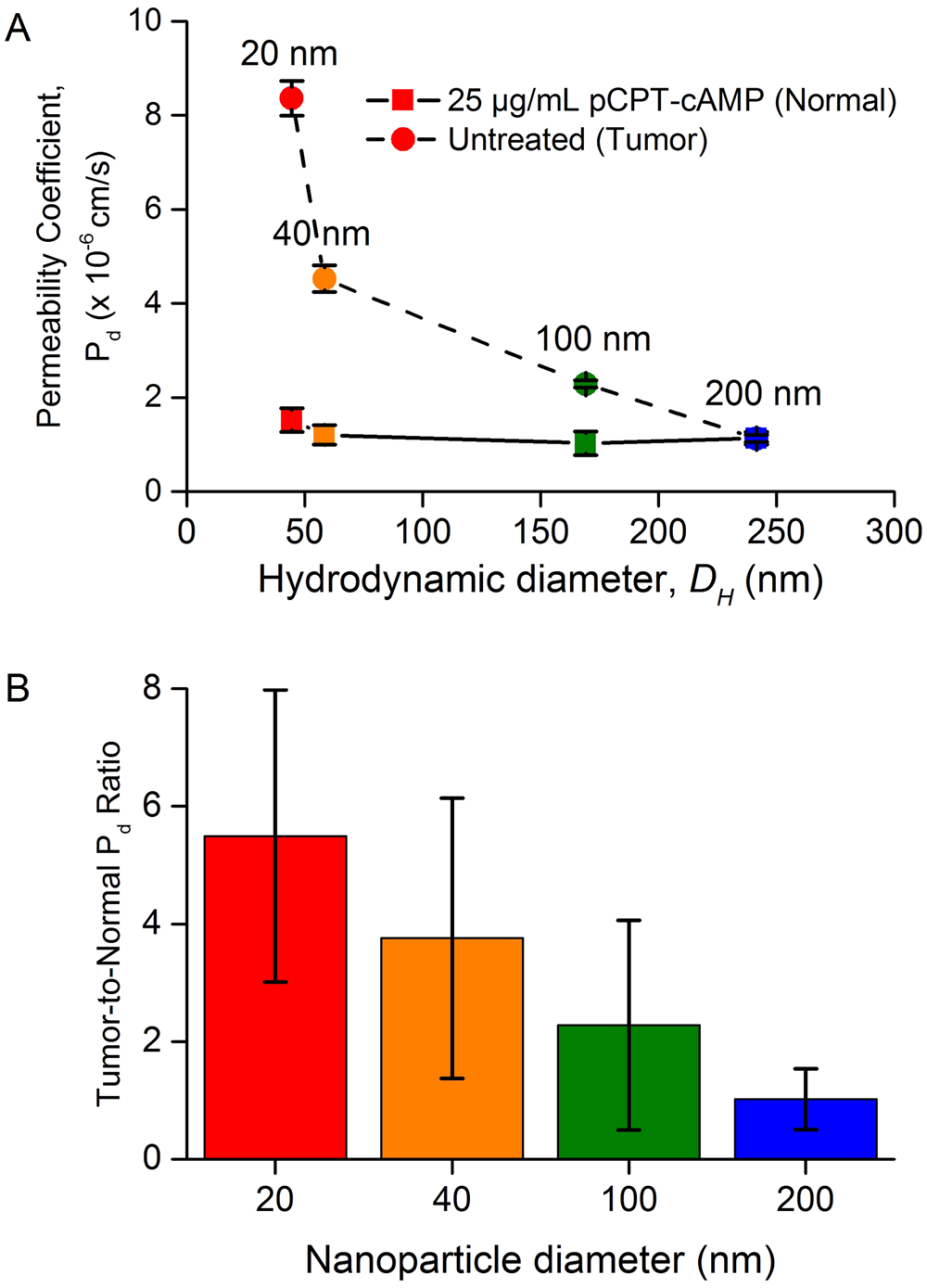
(**A**) Diffusional permeability, P_d_ of dextrans and pNPs against their hydrodynamic diameter in untreated and 25 µg/mL pCPT-cAMP treated microfluidic devices approximating leaky and normal healthy vasculature, respectively. With untreated microfluidic devices, the differences in P_d_ values of all pNPs were statistically significant (Student’s t-test. *p < 0.05). In the case of 25 µg/mL pCPT-cAMP treated devices, no statistically significant difference in P_d_ values was observed for any pNP size (Student’s t-test. *p < 0.05). (**B**) Ratio of untreated (tumor vasculature) to 25 µg/mL pCPT-cAMP treated (normal healthy vasculature) P_d_ values to represent the “tumor-to-normal” P_d_ ratio for all pNP sizes (Mean ± SEM).

We probed 20, 40, 100, and 200 nm pNPs for their P_d_ across untreated and 25 µg/mL pCPT-cAMP treated endothelial monolayers in microfluidic devices (Fig. 5A). Here, endothelial permeability was determined by two main routes: transcellularly through the cell via transcytosis, and paracellularly via the inter-endothelial junctions^52^. The P_d_ values of pNPs across untreated leaky endothelial monolayers decreased with increased particle size, following a non-linear curve (Fig. 5A), similar to that observed *in vivo* for the microvessels of skeletal muscle^38, 64^.

Furthermore, the P_d_ values of pNPs showed statistically significant difference between each size except between 100 and 200 nm pNPs (Fig. 5A) (p < 0.05). For smaller 20 and 40 nm pNPs, size dependent selectivity of the paracellular route was observed, which led to 20 nm pNPs being more permeable than 40 nm pNPs. As we approach the size limit of paracellular permeability in leaky vasculature, the difference in P_d_ between the larger 100 and 200 nm pNPs became insignificant, indicating limiting paracellular transport at these sizes. At these large sizes, the transcellular route became the dominant mode of transendothelial transport, which appeared to be less size-dependent.

To ensure that the differences in permeability were not attributed to the cytotoxicity of pNPs of different sizes, we examined the cell viability of HUVECs using PrestoBlue cell viability reagent and found that pNPs of different sizes did not affect cell viability when compared to controls (data not shown). This suggests that the differences observed in the P_d_ values between different sized pNPs were not due to cytotoxic effects of pNPs on HUVECs.

In the monolayers treated with 25 µg/mL pCPT-cAMP, the same set of pNPs experienced lower P_d_ across pCPT-cAMP treated endothelial cells for all sizes compared to untreated endothelial cells (Fig. 5A). The permeability was also comparably size-independent. The lower P_d_ is attributed to the shutdown of paracellular route with cAMP treatment, leaving the transcellular route as the dominant mode of transendothelial transport, which was also observed to be size independent across the size range of pNPs used in this study. The application of pCPT-cAMP has been shown in literature to increase cell-cell and cell-matrix tethering, reduce isometric tension development and also decrease inter-cellular gap formations^18, 60, 61^. Physiologically, cAMP is also known to mediate endothelial permeability via cAMP dependent protein kinase A (PKA)^61, 69–73^ and PKA independent mechanisms^74–76^. The induction of cAMP in endothelial cells activates PKA which in turn inhibits the activation of protein substrates RhoA^77^ and MLCK^78, 79^. As a result, the paracellular permeability is reduced by preventing actin-myosin cytoskeletal contractions that destabilize adheren junctions^52, 80^.

cAMP has also been shown to promote the expression of adherens and tight junction proteins such as ZO-1, VE-Cadherin, CLDN5, and Jam-A^19, 39, 60–62^. Together, these stabilize the inter-endothelial junctions between endothelial cells, thereby reducing paracellular permeability.

This reduction in paracellular permeability is significant because migration of macromolecules of diameters >3 nm (approximately the size of serum albumin) across the continuous endothelium will be hindered in intact healthy microvessels, thereby leaving transcellular vascular pathways being the dominant route responsible for their transport across the endothelium^81–86^ as observed in our treated case. *Ex vivo* experiments have shown that transcellular endocytotic processes result in P_d_ values that are less size dependent for the same diffusing NP^38, 44^. This similar size-invariant trend was also observed with cAMP-treated endothelial cells (Fig. 5A), where differences in P_d_ and hence size selectivity amongst all pNPs were not statistically significant. This suggests that with 25 µg/mL pCPT-cAMP HUVECs, pNPs of all sizes tested traversed the endothelial monolayer transcellularly as described earlier.

The obtained P_d_ values and non-linear inverse relationship between P_d_ and NP sizes observed in both untreated and cAMP-treated HUVECs culture show similarity to that of similar macromolecules observed *in vivo* for tumor and normal vasculature, respectively^38, 44^. This suggests a promising use of the microfluidic model of vascular-tissue interface as a physiologically relevant model for a systematic and quantitative study of the extravasation of nanomedicine-based systems for tumor delivery. In fact, this microfluidic model could potentially be used as platform to characterize a wide range of drugs, macromolecules or other particulates for transendothelial migration beyond this study on nanoparticles.

Here, it may be worth highlighting that our present study solely determines the passive diffusional permeability of the NPs across the endothelium, and did not account for differences in Starling forces between blood hydrostatic pressure and tissue interstitial fluid pressure, which may also set up a convective transport component to drive the NPs across the endothelium^36, 83^. While this does not compromise our diffusional permeability values obtained which are close to *in vivo* levels, the design of our microfluidic model does allow for a differential pressure to be set up between opposite media channels (Fig. 1A). This differential pressure would then allow user tunability of the pressure difference between the cell lumen and the gel channel for modelling different degrees of Starling forces. This is an added feature of the model, and one that helps to motivate the need for microfluidics.

### “Tumor-to-normal” permeability ratio

The P_d,tumor_ values of pNPs across untreated endothelium approximating the permeability of tumor vasculature showed greater size selectivity compared to the relatively size-invariant P_d,normal_ in cAMP-treated endothelium approximating the permeability of healthy vasculature (Fig. 5a). Based on this difference in size selectivity between tumor and healthy vasculature, we defined a “tumor-to-normal” P_d_ ratio, TNR = P_d,tumor_/P_d,normal_ to probe the selectivity of leaky vasculature over healthy vasculature for a range of different sizes of pNPs. Here, the TNR decreased from 5.49 to 1.02 as the size of pNPs increased from 20 to 200 nm (Fig. 5b). Such a decreasing trend demonstrates that the smaller pNPs tend to preferentially cross the endothelium monolayer of leaky vasculature compared to healthy vasculature much more than larger pNPs^87–89^. This means that as the size of pNP increases, the increase in P_d_ due to the leakier tumor vasculature decreases, and the TNR approaches unity with no difference in P_d_ between tumor and normal vasculature for large NPs. Therefore, the microfluidic model of vascular-tissue interface also allows us to characterize *in vitro* the selectivity of tumor vasculature to NPs.

## Conclusion

We have reported a relevant *in vitro* microfluidic model of vascular-tissue interface that can be used to provide a quantitative study of the permeability coefficients of NPs across the vascular endothelium under dynamic flow conditions. This model also allows facile systematic tunability of vascular endothelial permeability and thereby control over the transendothelial route taken by the NPs. The model was able to approximate physiologically relevant P_d_ values similar to *in vivo* values, that allowed us to conduct a systematic and quantitative evaluation of the extravasation rate of nanomedicine-based systems for tumor targeting. This can potentially be used to complement animal models as a facile screening tool in screening libraries of NPs prior to detailed animal studies, where the use of large scale animal models and their *in vivo*-associated complexities in controlling vasculature properties are not feasible.

We would also like to add that it would be too ambitious for us to conclude here that the untreated and cAMP-treated endothelium represents the tumor and normal vasculature in its entirety as many other factors that are related to the microenvironment are not represented in this model. Furthermore, the trans-endothelial transport of NPs *in vivo* is also influenced by hydrostatic pressure of blood and tumor interstitial fluid pressure *in vivo*, resulting in convective components that also drive NPs across the endothelium on top of passive diffusion^38, 90^.

Despite the limitations, we can still conclude that this platform allows for probing of endothelial permeabilities close to *in vivo* levels for different NP configurations. Apart from HUVECs, other endothelial barriers (e.g. alveolar endothelial cells as a model for the lungs^91^, COCA-2 for epithelial cells of the digestive tract^92^ or the blood brain barrier^93^) suitable for transport studies could potentially be implemented within this model. Besides, a wide range of NPs that have colorimetric (e.g. gold) or fluorescent properties could be characterized with the methodology presented here. This platform could therefore potentially offer valuable insights towards optimizing the design of NPs for a myriad of biomedical applications, including tumor drug delivery and would serve as a useful tool for the nanomedicine community.

## Electronic supplementary material

Supplementary Information
