# Supplementary material for: A Facile Method to Probe the Vascular Permeability of Nanoparticles in Nanomedicine Applications
Source: Sci Rep. 2017 Mar 31;7:707. doi: 10.1038/s41598-017-00750-3 (PMC5429672; doi:10.1038/s41598-017-00750-3)
Supplement: Supplementary file 1 — Supplementary Information [file 41598_2017_750_MOESM1_ESM.doc]

**Supplementary Information**

**A Facile Method to Probe the Vascular Permeability of Nanoparticles in Nanomedicine Applications**

*Yan Teck Ho^1^, Giulia Adriani^2^, Sebastian Beyer^2,3^, Phan-Thien Nhan^1,4^, Roger D Kamm^2,5*^, James Chen Yong Kah^1,6 **^*

^1^NUS Graduate School for Integrative Sciences and Engineering, National University of Singapore

^2^BioSyM Interdisciplinary Research Group, Singapore-MIT Alliance for Research and Technology, Singapore

^3^Federal Institute for Materials Research and Testing, Germany

^4^Department of Mechanical Engineering, National University of Singapore, Singapore

^5^Department of Biological Engineering and Department of Mechanical Engineering, Massachusetts Institute of Technology, USA

^6^Department of Biomedical Engineering, National University of Singapore, Singapore

CORRESPONDING AUTHOR

[*rdkamm@mit.edu](mailto:*rdkamm@mit.edu)

[**biekahj@nus.edu.sg](mailto:**biekahj@nus.edu.sg)

**Diffusion Coefficient of 200 nm corona-coated pNPs in the Central Gel Region**

Based on the fluorescent images from the time lapse imaging, we attempted to quantify the diffusion coefficient of the 200 nm corona-coated pNPs in the fibrin gel using Fick’s second law of diffusion:


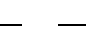


But since the point concentration of the pNPs was proportional to the fluorescence intensity at that point, the equation simplified to:


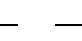


where
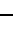
 refers to the change in intensity with time for a particular point within the gel region;
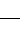
 refers to the second derivative of the change in fluorescence intensity of the nanoparticle at the same particular point moving towards the inner gel region (Figure 2); and D is the diffusion coefficient of the nanoparticles within the gel region.

By determining
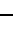
 and
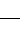
 from the timelapse images for a particular point within the gel region, we obtained an approximate diffusion coefficient of ≈ 2.04 x 10^-9^ cm²/s for the 200 nm pNPs in the fibrin gel. While the value was small, this non-zero value suggested that the pore size of fibrin gel was not the limiting factor in the permeability measurements of the nanoparticles.

Furthermore, our fluorescent time-lapse images of the largest 200 nm corona coated pNPs also showed the nanoparticles diffusing into the gel in both untreated and pCPT-cAMP treated HUVECs, with the diffusion front of the NPs readily diffusing through the fibrin gel (Figure S1 below). While it is true that the diffusion front of the 200 nm pNPs maintained a shorter distance compared to the smaller pNPs after the same time period (video not shown), this was expected since the 200 nm pNPs are larger in size. Hence, based on our empirical observation and P_d_ calculation, we did not think that the gates (labeled as blue in Figure 2A of the manuscript) may be blocked by nanoparticles with large sizes (e.g. 200 nm), which may further delay the diffusion of nanoparticles across the HUVEC monolayer into the central gel.


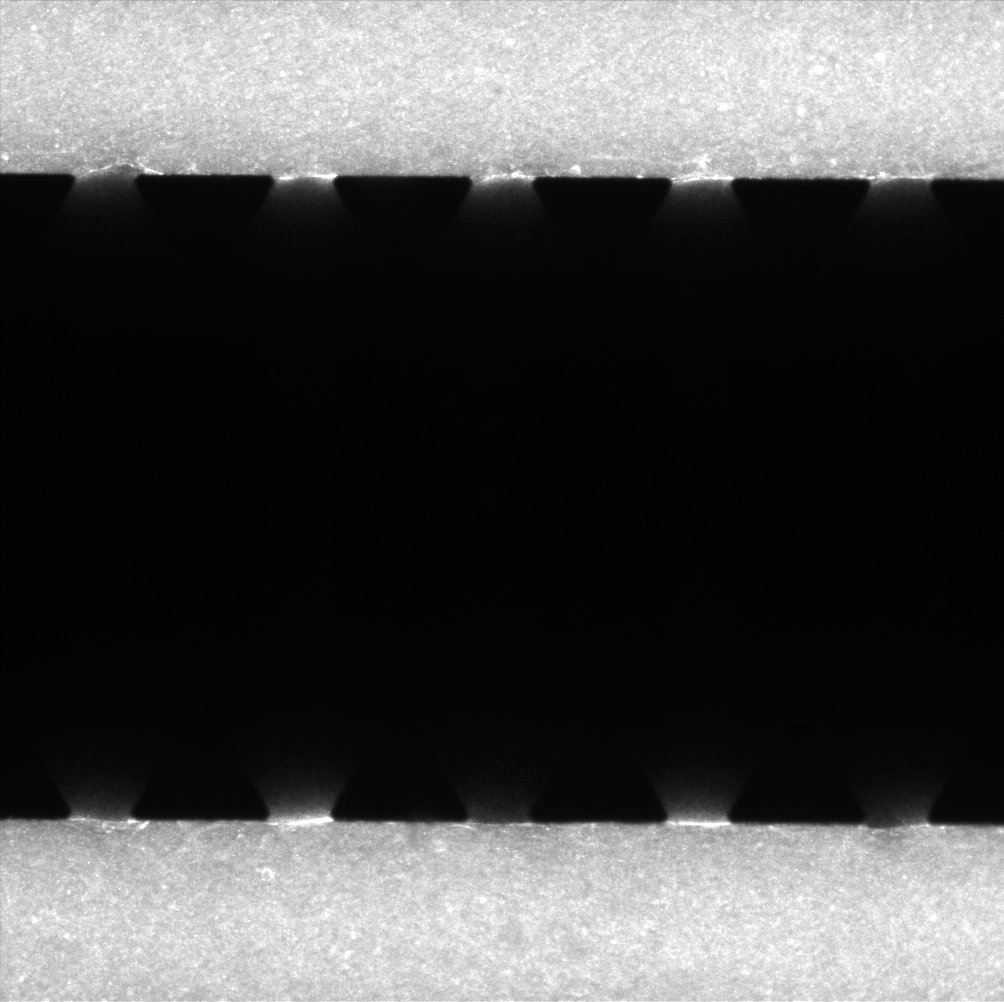


**Figure S1. Diffusion of 200 nm corona-coated pNPs into the gel in untreated HUVECs after 45 mins of introducing the fluorescent pNPs into the channel.**
